# Obscure Gastrointestinal Bleeding Due to Non-steroidal Anti-inflammatory Drug-Induced Colopathy

**DOI:** 10.7759/cureus.20278

**Published:** 2021-12-08

**Authors:** Huda A Meshikhes, Mohammed A Duhaileb, Ali A Alzahir, Sami A Almomen, Abdul-Wahed N Meshikhes

**Affiliations:** 1 Medicine, Immam Abdulrahman Bin Faisal University, Dammam, SAU; 2 Surgery, King Fahad Specialist Hospital, Dammam, SAU; 3 Gastroenterology and Hepatology, King Fahad Specialist Hospital, Dammam, SAU; 4 Surgery, Alzahra General Hospital, Qatif, SAU

**Keywords:** gastrointestinal hemorrhage, ulcers, intraoperative enteroscopy, enteropathy, non-steroidal antiinflammatory drugs

## Abstract

Obscure gastrointestinal (GI) bleeding poses a diagnostic challenge and is associated with high mortality. We report a case of life-threatening obscure GI bleeding precipitated by the ingestion of a non-steroidal anti-inflammatory drug (NSAID). The source of bleeding could not be identified preoperatively, and hence exploratory laparotomy was performed. An ileocaecal resection was undertaken based on the findings of the intraoperative enteroscopy. However, the bleeding recurred and repeated endoscopy examination identified the source to be multiple NSAID-induced ulcers that were scattered in the colo-rectum. The bleeding stopped spontaneously after a period of intensive supportive therapy and sulphasalazine enemas. This case highlights the diagnostic challenge of obscure GI bleeding. It also highlights the potentially life-threatening danger of GI bleeding secondary to NSAID-induced colopathy, even after a short course of treatment.

## Introduction

Obscure gastrointestinal (GI) bleeding continues to pose a major challenge in diagnosis and management. This is attributed to the inability to identify the source of bleeding which is either missed or overlooked during the index investigations. This subsequently leads to recurrent or persistent bleeding that requires repeated hospitalization, frequent investigations, and multiple transfusions [1]. Chronic ingestion of non-steroidal anti-inflammatory drugs (NSAIDs) is known to cause upper GI ulcers which may either bleed or perforate. Long-term ingestion of NSAIDs is also commonly reported to cause NSAID-induced enteropathy and to a lesser extent NSAID-colopathy [2]. However, bleeding rarely occurs after a short course of NSAID administration. We report here a case of GI bleeding from colo-rectal ulcers as a result of NSAID-induced colopathy following the ingestion of only two doses of NSAID.

## Case presentation

**Figure 1:**
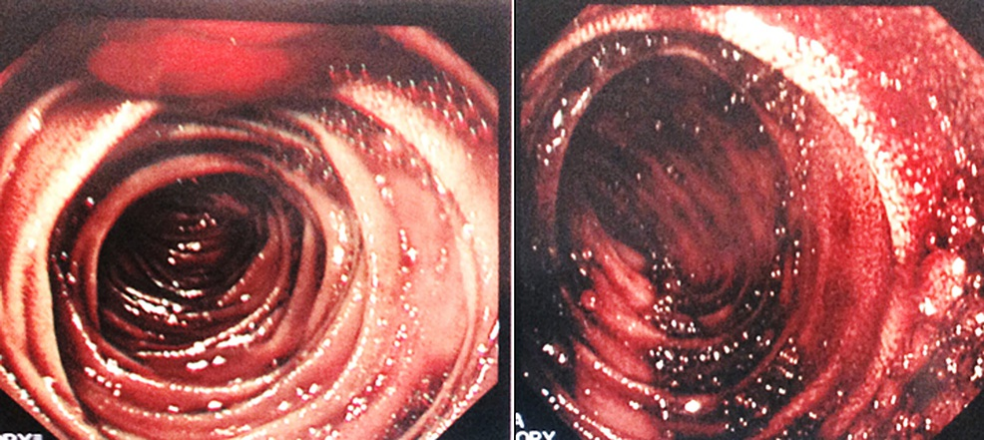
Endoscopic view of intraoperative enteroscopy revealing evidence of fresh bleeding, but no visible ulceration.

A 55-year-old male presented to a nearby hospital with a three-day history of melaena after *taking *two doses of an NSAID (diclofenac 50mg P.O.) for acute backache. He was known to suffer from hypertension and gastroesophageal reflux disease (GERD). He was managed conservatively with intravenous fluids and blood transfusion, and an upper GI endoscopy showed gastritis with no active bleeding. The patient left against medical advice and presented to our facility the next day in a state of shock after repeated episodes of bleeding per rectum. He was cold and clammy with tachycardia and hypotension (Pulse: 129 beats/min, BP: 87/60 mmHg). The abdomen was soft, lax, and non-tender, but the rectal examination revealed fresh blood. He was resuscitated aggressively and started on pantoprazole infusion. Routine blood tests showed low hemoglobin (5g/dL), normal serum electrolytes, and normal renal and liver profiles. As gastroscopy has already been done, colonoscopy was performed but visualization was poor due to the presence of blood clots and, therefore, the source of bleeding could not be identified. Red blood cell-labeled isotope scan was also done, but no identifiable source was visualized. On day 2, a computed tomography (CT) scan showed hyper-intensity of the proximal jejunal wall raising the possibility of bleeding. However, selective mesenteric angiography failed to demonstrate any bleeding source. Upper GI endoscopy was repeated showing evidence of gastritis, but no active gastro-duodenal bleeding. The patient continued to pass fresh blood per rectum needing seven units of packed RBCs transfusion. The hemoglobin remained low at 8.4g/100dL. As the bleeding was continuing and the preoperative investigations failed to localize the bleeding site, the decision was made to intervene surgically. Exploratory laparotomy revealed the presence of blood in the distal terminal ileum, and the right colon was full of clots. There was no obvious serosal pathology in the entire small bowel and colon. Intraoperative enteroscopy (IOE) showed fresh blood in the ileocecal region with multiple superficial mucosal ulcerations (Figure 1).

**Figure 2:**
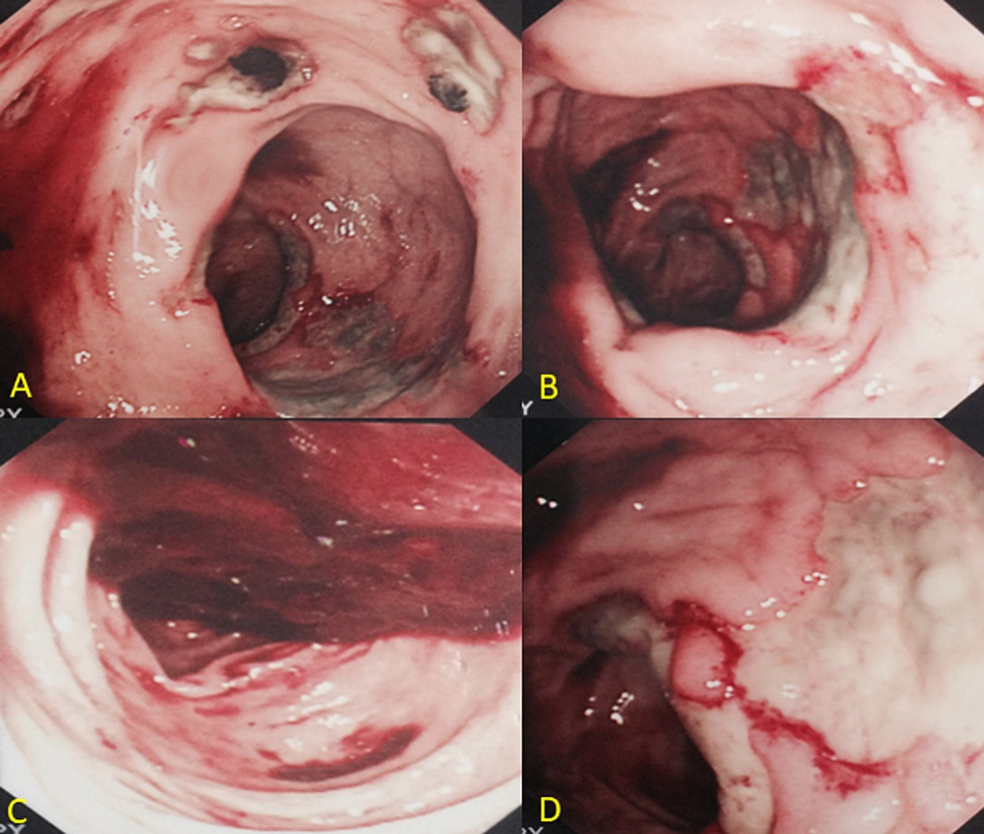
Endoscopic views of the colon via the mucus fistula showing multiple superficial and deep ulcerations especially in the rectum (A, B), with blood clots (C). Less frequent ulcers were seen in the colon (D).

Ileocecal resection was performed and the ileal and colonic ends were brought out as an end-ileostomy and a mucous fistula, respectively. The histopathology of the resected ileocecal specimen showed scattered ulcers in the terminal ileum with serositis. On the third postoperative day (POD 3), blood was noticed coming through the mucus fistula in the colostomy bag. Colonic lavage was done through the mucus fistula, and a lot of dark blood and clots were evacuated. Colonoscopy (through the mucus fistula) showed multiple deep and superficial ulcers scattered in the descending colon and rectum (Figures 2A-2D).

Multiple biopsies were taken and showed colonic mucosa with ulcerations and regenerative changes. Immunostaining for cytomegalovirus (CMV) was negative. Blood was sent for vasculitis workup, but the anti-nuclear antibodies (ANA) and anti-DNA came back negative. The patient was started on sulphasalazine enemas and his condition improved with no further bleeding episodes. He was discharged home after a total hospital stay was 21 days.

**Figure 3:**
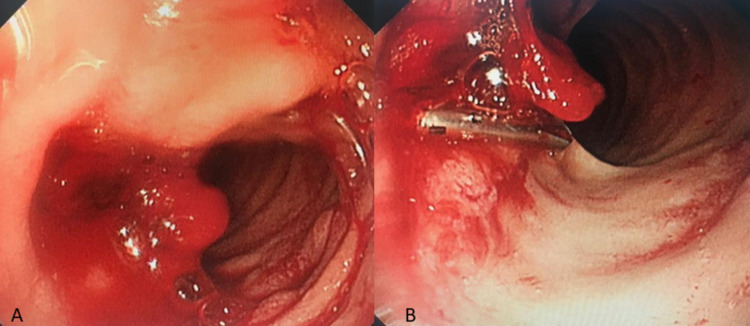
Endoscopic view showing a bleeding ulcer in the second part of the duodenum (A). It was treated by endoscopic clipping (B).

Six weeks later, he presented to the emergency department with a history of bleeding via the ileostomy. Upper GI endoscopy revealed a bleeding duodenal ulcer (Figures 3A, 3B). The bleeding was controlled endoscopically, and he was discharged home on oral pantoprazole 40 mg daily.

Six months later, he was prepared for the reversal of his stomas. Preoperative upper and lower endoscopy and capsule enteroscopy were normal with no apparent GI pathology. He underwent exploratory laparotomy, division of adhesions, and ileocolic anastomosis. The patient was discharged five days later and remained well at a 36-month follow-up.

## Discussion

Obscure bleeding accounts for 5% of all GI bleeding, and the small bowel is the source of bleeding in about 75% of cases [1,3]. It was defined by the American Gastroenterological Association as bleeding from the GIT that persists or recurs without an obvious etiology after an upper endoscopy, colonoscopy, and radiologic evaluation of the small bowel [4]. The identification of the underlying etiology often remains elusive despite extensive evaluations, resulting in repeated admissions multiple transfusions, and frequent endoscopic examinations (upper and lower GI endoscopies) in some cases [5]. Failure to identify the source of bleeding necessitates the use of capsule enteroscopy; this was done in this patient, but images could not be retrieved because of technical failure. Also, in this case, the bleeding rate was slow and intermittent making identification of the bleeding source by either angiography or bleeding scan difficult. A challenging issue in obscure GI bleeding is that the small bowel is not as readily accessible for endoscopic examination as the gastro-duodenum and colo-rectum. However, its examination can now be performed using several radiologic and endoscopic modalities as well as surgical approaches which include exploratory laparoscopy/laparotomy with and without IOE. The diagnostic yield of IOE is less than 90%, and bleeding recurs in up to 60% of patients because the pathology is often overlooked due to limited visibility [6,7]. IOE was performed in this case via enterotomy in an attempt to localize the source of bleeding and therefore, an ileocaecal resection was performed. However, bleeding recurred as IOE was not decisive in determining the exact site of the bleeding. IOE is not recommended in every case as it is associated with complications such as serosal tears, avulsion of mesenteric vessels, prolonged ileus, and perforation; thus it should be reserved only for selected patients who present with recurrent bleeds requiring repeated admissions and multiple transfusions after negative comprehensive radiological and endoscopic evaluation [8].

This case also highlights the dangers of NSAIDs in certain individuals; our patient started GI bleeding after only two doses of diclofenac. This may be considered as a type of “hypersensitivity” reaction which led to the development of multiple bleeding colorectal ulcers after a very short course of NSAID. This event is unprecedented as upper GI ulcers and complications; mainly bleeding and perforation develop after the chronic and extensive use of NSAIDs. Serious GI complications are experienced in 1%-2% of users during the course of NSAID treatment [2]. Moreover, NSAID-induced mucosal damage occurs more frequently in the small bowel (eneteropathy) and to a lesser extent in the large bowel (colopathy) in more than 60% of long-term NSAID users. In most cases, the damage is subclinical in the form of inflammation, erosions, ulceration, and iron-deficiency anemia. Nevertheless, short-term users of NSAIDs are by no means immune to serious complications [9]. Evidence of macroscopic injury to the small intestine after two weeks of slow-release diclofenac ingestion occurred in up to 68% of healthy volunteers, as was demonstrated by capsule enteroscopy [10]. This case developed life-threatening GI bleeding after administering only two doses of diclofenac for acute back pain.

The introduction of different enteroscopy modalities such as wireless capsule endoscopy and double-balloon enteroscopy has allowed visualization of NSAID-induced bowel lesions. The injuries may be recognized by their endoscopic characteristic features as seen in Figures 2A-2D. They are usually multiple superficials to deep ulcers with an irregular arrangement and <1 cm in diameter) [11]. CT and MRI are also helpful in diagnosing NSAID-induced small bowel diaphragms and strictures where the use of wireless-capsule enteroscopy is contraindicated [12].

 In recent years, there is a noticeable declining trend in hospitalizations due to NSAID-induced upper GI complications as a result of the concomitant administration of gastro-protective drugs. On the contrary, there is a significant increase in lower GI tract complications as a result of NSAID enteropathy [2]. Recent studies suggest that proton pump inhibitors (PPIs) and other commonly used drugs for protecting the gastro-duodenum do not offer the same beneficial protective effect to the lower GI tract. Moreover, they may significantly worsen NSAID-induced damage in the small and large intestines by altering the intestinal microbiota and hence implicated in increasing the risk of lower GI bleeding [13]. Furthermore, the increasing use of enteric-coated and slow-release NSAID may be considered as a contributory factor of NSAID-colopathy, which is more commonly affecting the right side of the colon. This fact argues for the direct local rather than the systemic effect of the drug. In the present case, the ulcers were mainly in the descending colon and rectum. This argues against the direct local effect theory [14].

When NSAID entero-colopathy occurs, it is more difficult to treat than that of the upper GIT, as there is no proven effective treatment therapy. The treatment focuses mainly on the withdrawal of the causative drug [15]. Antibiotics and sulphasalazine have been proved to be effective in animals, but they have not been properly tested in humans. In this case, sulphasalazine enemas were given via the mucus fistula which may have helped ulcer healing. Also, selective COX-2 inhibition is emerging as a potential alternative to NSAIDs in the reduction and prevention of damage in the lower GI tract in rheumatologic patients [16]. However, its long-term safety needs to be further substantiated.

This case highlights the importance of keeping in mind the diagnosis of NSAID-induced enteropathy/colopathy in any patient presenting with obscure GI bleeding even after a short history of NSAIDs ingestion.

## Conclusions

The diagnosis and management of obscure GI bleeding remain a challenge. It demands frequent admissions, repeated endoscopic and radiological investigations as well as multiple blood transfusions. The incidence of NSAID-induced injury to the lower GIT is on the increase with a good proportion of patients may be presenting with obscure GI bleeding. Entero-colopathy should be considered a source of obscure GI bleeding even after a short course of NSAID treatment. Once the diagnosis is made, treatment is by supportive therapy and cessation of the causative drug. The search for an effective prevention strategy is continuing. Unfortunately, most of the available evidence on the incidence and danger of NSAID entero-colopathy is based on animal studies and sporadic case reports in the absence of large randomized studies.
